# Students’ approaches to medical school choice: relationship with students’ characteristics and motivation

**DOI:** 10.5116/ijme.5921.5090

**Published:** 2017-06-12

**Authors:** Anouk Wouters, Gerda Croiset, Nienke R. Schripsema, Janke Cohen-Schotanus, Gerard W.G. Spaai, Robert L. Hulsman, Rashmi A. Kusurkar

**Affiliations:** 1 VUmc School of Medical Sciences, Research in Education, Amsterdam, the Netherlands; 2 Center for Education Development and Research in Health Professions (CEDAR), University of Groningen the Netherlands; 3 Center for Research and Innovation in Medical Education, University of Groningen and University Medical Center Groningen, Groningen, the Netherlands; 4 Center for Evidence-Based Education, AMC-UvA, Amsterdam, the Netherlands; 5 Department of Medical Psychology, AMC-UvA, Amsterdam, the Netherlands

**Keywords:** Admissions, selection, motivation, self-determination theory, the Netherlands

## Abstract

**Objectives:**

The aim was to examine main reasons for students’ medical school choice and their relationship with students’ characteristics and motivation during the students’ medical study.

**Methods:**

In this multisite cross-sectional study, all Year-1 and Year-4 students who had participated in a selection procedure in one of the three Dutch medical schools included in the study were invited to complete an online survey comprising personal data, their main reason for medical school choice and standard, validated questionnaires to measure their strength of motivation (Strength of Motivation for Medical School-Revised) and autonomous and controlled type of motivation (Academic Self-regulation Questionnaire). Four hundred seventy-eight students participated. We performed frequency analyses on the reasons for medical school choice and regression analyses and ANCOVAs to study their associations with students’ characteristics and motivation during their medical study.

**Results:**

Students indicated ‘city’ (Year-1: 24.7%, n=75 and Year-4: 36.0%, n=52) and ‘selection procedure’ (Year-1: 56.9%, n=173 and Year-4: 46.9%, n=68) as the main reasons for their medical school choice. The main reasons were associated with gender, age, being a first-generation university student, ethnic background and medical school, and no significant associations were found between the main reasons and the strength and type of motivation during the students’ medical study.

**Conclusions:**

Most students had based their medical school choice on the selection procedure. If medical schools desire to achieve a good student-curriculum fit and attract a diverse student population aligning the selection procedure with the curriculum and taking into account various students’ different approaches is important.

## Introduction

Medical schools aim to admit students who are motivated and fit with their curriculum best so that students can perform optimally.^1^^,^^2^ When students apply, however, they may focus on other characteristics of the medical schools besides the curriculum when choosing to which schools to apply. Moreover, different types of students may have varying approaches. Students’ approaches have thus far gained little attention. In this study, we investigate the reasons for students’ medical school choice and their association with student characteristics and motivation during students’ medical study.

Little is known about what drives applicants’ medical school choice. Research on this topic has been conducted mainly in the UK (and one study from the US). The reasons indicated as the common ones for choosing a medical school are the curriculum (e.g. teaching and course characteristics),^3^^-^^7^ school (e.g. reputation and atmosphere),^3^^,^^5^^-^^8^ admissions (e.g. the interview process),^4^^,^^5^ and geographical characteristics (e.g. city).^3^^-^^7^ The contexts in which these studies have been conducted are similar to each other with respect to the fact that applicants are able to apply to multiple medical schools at once before they participate in medical school selection (i.e. from up to four schools in the UK to as many as applicants want in the US, with an average of 15 per applicant).^9^ They make their final choice after they receive admissions offers from one or more medical schools. In such situations, applicants may make their first choices based on the selection procedures of the medical schools and their final choice, after being offered admission, based on the curriculum and school characteristics. When applicants are restricted to choosing one medical school, and they have to choose before entering any selection procedure, as is the case in the Netherlands, the selection procedure may play a larger role in their choices. How applicants make their medical school choice in such a setting has not yet been investigated. Because of the creation of this unique situation in the Netherlands, conducting this investigation in this context may also provide initial insights into how students make their first choices in other contexts, such as the UK or US.

The Dutch admissions system is gradually changing from admission based on pre-university grade point average (GPA; i.e. top pre-university GPA and weighted lottery) and selection procedures to admission based on selection only. Admission based on pre-university GPA is regulated at the national level, while admission based on selection is regulated at the institutional level.^10^ Students can choose to apply through one or both routes. Applicants with a top pre-university GPA (≥8 out of 10) are admitted at the medical school of their choice. Applicants with lower GPAs enrol in the weighted lottery and indicate their top 3 medical school preferences. In addition, applicants are allowed to apply to a selection procedure at one medical school per year. Each medical school can design its own selection procedure. This allows the medical schools to select the students they consider most likely to perform well in their medical programmes. When rejected in selection, applicants are automatically enrolled in the weighted lottery. Over the years, the proportion of places that medical schools are allowed to fill through selection has increased from a maximum of 50% in 2005 to a maximum of 100% in 2013 onwards. This transition enables the investigation of applicants’ behaviour in different situations.

Few studies have investigated the relation between the reasons for students’ medical school choice and students’ characteristics. In a Scottish study, females valued course aspects more than males, and location was more important for younger students than for older students.^6^ Compared to students born within the UK, reputation and prestige were more important reasons, and course aspects and location were less important reasons for those born outside the UK. Among American students, ethnic minority students placed greater emphasis on diversity aspects in selecting their medical school. Moreover, reasons differed between students from different medical schools.^7^ All Asian females in a UK interview study indicated the importance of the location of the medical school because of their wish to live at home during their medical study.^5^

To achieve the best learning outcomes, a high level of motivation alone is not sufficient. Research shows that the type of motivation is more important than the strength of motivation.^11^^,^^12^ Self-determination theory (SDT) acknowledges the differences in the quality of motivation and describes motivation along a continuum.^13^ Students can lack motivation or be motivated based on external factors or internal factors. Intrinsic motivation (e.g. a sincere interest in an activity) and identified regulation (e.g. a positive valuation of an activity) together form what is called autonomous motivation. Introjected regulation (internal pressures, such as feelings of shame or guilt) and external regulation (external pressures, such as status or parental pressure) together form what is called controlled motivation. Motivation is considered dynamic and can change from controlled to autonomous, and vice versa.^14^ Autonomous motivation is considered the most desirable type of motivation because it is associated with better learning outcomes and positive well-being of students.^15^^-^^20^ Moreover, students with autonomous motivation are more likely to deliver autonomy supportive patient care, which benefits health care.^11^ If medical schools want to attract students that fit best with their curriculum, they should try to attract students with autonomous motivation for choosing their curriculum.

The reasons for students’ medical school choice have not yet been investigated in relation to students’ motivation during their medical study. The aims of this study were to investigate the main reasons students choose a particular medical school, whether different student characteristics are associated with different reasons for their medical school choice and whether the reasons are associated with the strength and type of motivation during their medical study. We examined this among students of three of the eight Dutch medical schools, i.e. VUmc School of Medical Sciences Amsterdam (VUmc), Academic Medical Center Amsterdam (AMC) and University Medical Center Groningen (UMCG), to detect possible medical school effects. The medical study in the Netherlands consists of three years of pre-clinical education followed by three years of clinical education, after which students obtain their medical degree. The curricula across the Netherlands are largely comparable because they are all vertically integrated, student-centred and driven by nationally standardized end terms.^10^^,^^21^ Yet, local differences between the medical schools exist, such as the focus of education (problem-based versus theme-based), student intake and proportion of students admitted through selection. An overview of the medical schools’ characteristics is provided in Table 1.

The following research questions guided our study:

       1. What are students’ main reasons for applying to a particular medical school?

       2. Are the main reasons for students’ medical school choice associated with students’ characteristics (i.e. gender, age, ethnic background, being a first-generation university student, having a parent in the medical profession, area of growing up, medical school)?

       3. Are the main reasons for students’ medical school choice associated with their motivation for medical study during their first and fourth year of the medical programme?

**Table 1:** Medical school characteristics

Med School	VUmc	AMC	UMCG
Focus of the curriculum	Theme-based	Theme-based	Problem-based
Selection procedure	*Selection procedure A^22^* Two phases: 1) Portfolio including previous academic records and extracurricular activities. 2) Lectures followed by assessment of academic skills, measured with tests about medical subjects and study skills.	*Selection procedure B^23^* Two phases: 1) Cognitive tests and portfolio including previous academic records and extracurricular activities. 2) Lecture followed by an academic test and three-station MMI (Year-1) or interview (Year-4).	*Selection procedure C^24^* Two phases: 1) Portfolio (comprising sections on pre-university education, extracurricular activities, and reflection) and academic and non-academic tests. 2) Patient lecture followed by assignments (related to the lecture, writing an essay, and scientific reasoning) and a four-station MMI assessing communication skills, collaboration skills and reflection.
Student intake and places assigned through selection	Year-1: 60% of 350 places Year-4: 50% of 350 places	Year-1: 75% of 350 places Year-4: 50% of 350 places	Year-1: 100% of 410 places Year-4: 50% of 410 places
Geographical location	In the capital city (Amsterdam)	In the capital city (Amsterdam)	In a smaller city in the North of the country

## Methods

### Study design

This was a multisite cross-sectional study using an online survey (Net Questionnaire) comprising personal data, a multiple-choice question on students’ main reasons for their medical school choice and standard, validated questionnaires to measure motivation.

### Study participants

In the academic year 2013-2014, Year-1 and Year-4 students were invited via e-mail (with up to two reminders) to participate in this study. For every 10 participants, a gift card of €25 was rewarded through random selection. Participation was voluntary, and informed consent was obtained from all participants. The data were anonymized before analyses. The study was approved by the Ethical Review Board of the Netherlands Association for Medical Education (NVMO-ERB).

Students who had participated in a selection procedure (both accepted and rejected students) were included in this study. The 666 participants (response rate 35%) included 387 Year-1 students and 273 Year-4 students. To answer our research questions, we included only a subsample of students who had participated in the selection for the regular medical programme and had indicated a main reason for their medical school choice (n=478, 315 Year-1 students and 163 Year-4 students). Because the percentage of students the medical schools were allowed to admit through selection has increased from 50% to 100% over the years, we conducted the analyses for the Year-1 and Year-4 subsamples separately. The mean ages of the subsamples were 18.8 and 22.8 years old for Year-1 and Year-4, respectively. The gender distribution was comparable across the subsamples and representative of that in Dutch medical schools; 74.5% females (n=283) in Year-1 and 73.9% females (n=190) in Year-4.

### Data collection instruments

The survey contained one item about students’ main reason for their medical school choice.Based on the literature,^3^^-^^8^ we included the city, curriculum, university culture and selection procedure as response options. If none were applicable to students, they could choose ‘other’ and provide their main reason as an open comment in a textbox.

The 15-item Strength of Motivation for Medical School-Revised^25^^-^^27^ (SMMS-R) was used to measure students’ strength of motivation*.* Students had to indicate, on a 5-point Likert-scale, to what extent they agreed with the statements (1 = strongly disagree; 5 = strongly agree). We used the total scale score, as well as the scores on the subscales: Willingness to sacrifice (example item: ‘I would still choose medicine even if that meant I would never be able to go on holidays with my friends anymore’), Readiness to start (example item: ‘I wouldn’t consider any other profession than becoming a doctor’ and Persistence (example item: ‘I would quit studying medicine if I were 95% certain that I could never become the specialist of my choice’). The Cronbach’s alpha values for reliability for the (sub) scales Autonomous motivation, Controlled motivation, Strength of motivation, Willingness to sacrifice, Readiness to start and Persistence were 0.82, 0.84, 0.79, 0.64, 0.67 and 0.58, respectively.

**Table 2:** Distribution of main reasons for medical school choice across Year-1 and Year-4 students

Year-1		Main reason for choice of university			
		City	Curriculum	University culture	Selection procedure
Medical school	VUmc (n = 90)	20.0% (n = 18)	7.8% (n = 7)	10.0% (n = 9)	62.2% (n = 56)
	AMC (n = 92)	15.2% (n = 14)	5.4% (n = 5)	9.8% (n = 9)	69.6% (n = 64)
	UMCG (n=122)	35.2% (n = 43)	18.0% (n = 22)	3.3% (n = 4)	56.9% (n = 53)
Gender	Male (n = 70)	35.7% (n = 25)	8.6% (n = 6)	8.6% (n = 6)	47.1% (n = 33)
	Female (n = 234)	21.4% (n = 50)	12.0% (n = 28)	6.8% (n = 16)	59.8% (n = 140)
Ethnic background	Majority (n = 261)	24.1% (n = 63)	11.1% (n = 29)	6.9% (n = 18)	57.9% (n = 151)
	Western minority (n = 22)	31.8% (n = 7)	13.6% (n = 3)	9.1% (n = 2)	45.5% (n = 10)
	Non-Western minority (n = 21)	23.8% (n = 5)	9.5% (n = 2)	9.5% (n = 2)	57.1% (n = 12)
Having a medical doctor as a parent	Yes (n = 47)	31.9% (n = 15)	12.8% (n = 6)	8.5% (n = 4)	46.8% (n = 22)
	No (n = 256)	23.0% (n = 59)	10.9% (n = 28)	7.0% (n = 18)	59.0% (n = 151)
Being a first-generation university student	Yes (n = 55)	20.0% (n = 11)	18.2% (n = 10)	1.8% (n = 1)	60.0% (n = 33)
	No (n = 248)	25.8% (n = 64)	9.3% (n = 23)	8.5% (n = 21)	56.5% (n = 140)
Area of growing up	City (n =137)	27.0% (n = 37)	10.2% (n = 14)	8.0% (n = 11)	54.7% (n = 75)
	Village (n =167)	22.8% (n = 38)	12.0% (n = 20)	6.6% (n = 11)	58.7% (n = 98)
Total (n = 304)		24.7% (n = 75)	11.2% (n = 34)	7.0% (n = 22)	56.9% (n = 173)
Year-4		Main reason for choice of university			
		City	Curriculum	University culture	Selection procedure
Medical school	VUmc (n = 26)	19.2% (n = 5)	3.8% (n = 1)	3.8% (n = 1)	73.1% (n = 19)
	AMC (n = 35)	14.3% (n = 5)	14.3% (n = 5)	11.4% (n = 4)	60.0% (n = 21)
	UMCG (n = 84)	50.0% (n = 42)	14.3% (n = 12)	2.4% (n = 2)	33.3% (n = 28)
Gender	Male (n = 34)	32.4% (n = 11)	14.7% (n = 5)	5.9% (n = 2)	47.1% (n = 16)
	Female (n =111)	36.9% (n = 41)	11.7% (n = 13)	4.5% (n = 5)	46.8% (n = 52)
Ethnic background	Majority (n =130)	37.7% (n = 49)	9.2% (n = 12)	4.6% (n = 6)	48.5% (n = 63)
	Western minority (n=10)	20.0% (n = 2)	40.0% (n = 4)	10.0% (n = 1)	30.0% (n = 3)
	Non-Western minority (n = 5)	20.0% (n = 1)	40.0% (n = 2)	0.0% (n = 0)	40.0% (n = 2)
Having a medical doctor as a parent	Yes (n =15)	26.7% (n = 4)	20.0% (n = 3)	0.0% (n = 0)	53.3% (n = 8)
	No (n =130)	36.9% (n = 48)	11.5% (n = 15)	5.4% (n = 7)	46.9% (n = 68)
Being a first-generation university student	Yes (n = 31)	25.8% (n = 8)	9.7% (n = 3)	6.5% (n = 2)	58.1% (n = 18)
	No (n =113)	38.9% (n = 44)	12.4% (n = 14)	4.4% (n = 5)	44.2% (n = 50)
Area of growing up	City (n = 54)	24.1% (n = 13)	16.7% (n = 9)	5.6% (n = 3)	53.7% (n = 29)
	Village (n = 91)	42.9% (n = 39)	9.9% (n = 9)	4.4% (n = 4)	42.9% (n = 39)
Total (n = 145)		36.0% (n = 52)	12.4% (n = 18)	4.8% (n = 7)	46.9% (n = 68)

The Cronbach’s alpha values for the SMMS-R subscales are slightly below the desired value of 0.70. The findings should therefore be interpreted with caution.

The type of motivationwas based on the concept of motivation put forth by the SDT.^13^ Autonomous motivation and controlled motivation were measured with the 16-item Academic Self-regulation Questionnaire.^12^ Students had to indicate, on a 5-point Likert-scale, how important they considered the stated reasons for studying medicine (1= not important at all; 5=very important). An example item for autonomous motivation is: ‘I am motivated to study for the medical study because… I enjoy doing it’. An example item for controlled motivation is: ‘I am motivated to study for the medical study because… I want others to think I’m a good student’. The Cronbach’s alpha values for reliability of the scales Autonomous motivation and Controlled motivation were 0.82 and 0.84, respectively.

### Procedure

The survey was administered online using NetQuestionnaires. Data on the main reason for students’ medical school choice, strength of motivation, type of motivation,age, gender, medical school (VUmc/AMC/UMCG), being a first-generation university student (yes/no), whether a student had one or two parents working in the medical profession (yes/no), ethnicity (Dutch/Western minority/non-Western minority) and area of growing up (city/village) were collected as part of the questionnaire. Ethnicity was defined following the definition that states that a person belongs to an ethnic minority group if at least one of the parents was born outside the Netherlands.^28^

**Table 3:** Regression analyses of student characteristics and reasons for medical school choice

Student characteristic					
Year-1		Model 1 City OR (95% CI); p-value	Model 2 Curriculum OR (95% CI); p-value	Model 3 University culture OR (95% CI); p-value	Model 4 Selection procedure OR (95% CI); p-value
Female		**2.20** (1.16-4.15); 0.02	0.57 (0.20-1.64); 0.30	1.36 (0.48-3.82); 0.56	**0.56** (0.31-1.00); 0.05
Age ≥ 19 years		1.00 (0.55-1.80); 0.99	0.50 (0.21-1.17); 0.11	0.39 (0.13-1.13); 0.08	**1.69** (1.00-2.84); 0.05
Ethnic background					
	Majority	1.0	1.0	1.0	1.0
	Western minority	0.44 (0.16-1.24); 0.12	1.22 (0.25-5.99); 0.81	0.65 (0.13-3.24); 0.60	2.03 (0.78-5.23); 0.15
	Non-Western minority	0.82 (0.28-2.47); 0.73	0.89 (0.18-4.40); 0.89	0.73 (0.15-3.67); 0.70	1.28 (0.49-3.33); 0.62
Being a first- generation university student		1.56 (0.69-3.52); 0.29	**0.28** (0.11-0.70); 0.01	5.12 (0.66-40.04); 0.12	0.96 (0.50-1.84); 0.90
Having a medical doctor as a parent		0.76 (0.37-1.58); 0.46	0.58 (0.20-1.65); 0.31	1.06 (0.32-3.51); 0.93	1.52 (0.77-2.99); 0.23
Growing up in city		0.81 (0.46-1.43); 0.47	1.17 (0.53-2.59); 0.70	0.98 (0.39-2.43); 0.96	1.10 (0.67-1.80); 0.72
University^†^					
	UMCG	1.0	1.0	1.0	1.0
	VUmc	**2.43** (1.23-4.79); 0.01	**3.11** (1.19-8.16); 0.02	0.33 (0.10-1.14); 0.08	**0.41** (0.23-0.74); 0.00
	AMC	**3.43** (1.66-7.06); 0.00	**3.46** (1.19-10.03); 0.02	**0.24** (0.07-0.87); 0.03	**0.35** (0.19-0.64);0.00
Year-4		Model 1 City OR (95% CI); p-value	Model 2 Curriculum OR (95% CI); p-value	Model 3 University culture OR (95% CI); p-value	Model 4 Selection procedure OR (95% CI); p-value
Female		0.67 (0.30-1.90); 0.55	1.42 (0.41-4.93); 0.58	1.11 (0.17-7.20); 0.91	1.04 (0.44-2.47); 0.92
Age ≥ 23 years		1.21 (0.54-2.70); 0.65	1.70 (0.52-5.59); 0.38	0.73 (0.14-3.72); 0.70	0.71 (0.33-1.50); 0.37
Ethnic background					
	Majority	1.0	1.0	1.0	1.0
	Western minority	2.22 (0.38-13.00); 0.38	**0.12** (0.02-0.60); 0.01	0.30 (0.03-3.40); 0.33	3.40 (0.70-16.44); 0.13
	Non-Western minority	1.0 (0.07-13.44);1.00	**0.07** (0.01-0.74); 0.03	n/a	4.00 (0.49-32.65); 0.20
Being a first- generation university student		1.73 (0.64-4.70); 0.28	2.28 (0.40-12.99); 0.35	0.70 (0.11-4.28); 0.70	0.51 (0.20-1.29); 0.16
Having a medical doctor as a parent		1.77 (0.46-6.82); 0.41	0.84 (0.17-4.23); 0.84	n/a	0.45 (0.13-1.56); 0.21
Growing up in city		1.66 (0.71-3.89); 0.24	0.93 (0.28-3.05); 0.90	1.08 (0.18-6.46); 0.94	0.63 (0.28-1.39); 0.25
University^†^					
	UMCG	1.0	1.0	1.0	1.0
	VUmc	**4.72** (1.58-14.15); 0.01	3.57 (0.40-32.22); 0.26	0.66 (0.06-7.78); 0.74	**0.17** (0.06-0.48); 0.00
	AMC	**5.15** (1.72-15.46); 0.00	0.95 (0.24-3.79); 0.95	0.20 (0.03-1.37); 0.10	**0.39** (0.16-0.95); 0.04

Note: Numbers in bold denote significant ORs (p < 0.05)

OR = odds ratio; 95% CI = 95% confidence interval

n/a = not available (due to small group sizes)

*Year-1: VUmc compared to AMC (reference group): Model 1 City OR 0.71 (95% CI 0.31-1.61, p=0.41); Model 2 Curriculum OR 0.90 (95% CI 0.26-3.09, p=0.87); Model 3 University culture OR 1.35 (95% CI 0.46-3.92, p=0.59); Model 4 Selection procedure OR 1.16 (95% CI 0.60-2.25, p=0.65).

†Year-4: VUmc compared to AMC (reference group): Model 1 City OR 0.92 (95% CI 0.22-3.76, p=0.90); Model 2 Curriculum OR 3.73 (95% CI 0.35-39.50, p=0.27); Model 3 University culture OR 3.24 (95% CI 0.31-33.85, p=0.33); Model 4 Selection procedure OR 0.45 (95% CI 0.14-1.45, p=0.18).

### Data analysis

We calculated the frequencies and percentages for each main reason for students’ medical school choice. We assessed the associations between the main reasons and students’ characteristics using binary logistic regression to calculate odds ratios (ORs). The variable main reason for students’ medical school choice was transformed into dummy variables for analyses. ORs reflect the change in the probability of a choice based on that specific reason relative to the probability of a choice based on one of the other reasons associated with each of the independent variables (i.e. students’ characteristics). An OR of > 1 reflects an increased likelihood of a choice based on that specific reason compared to a choice based on one of the other reasons. To investigate the association between the reasons for students’ medical school choice and students’ motivation during their medical study, we conducted analyses of variance while controlling for age and gender (ANCOVA). Bonferroni post-hoc analyses were used to correct for multiple comparisons. Analyses were performed using IBM SPSS Statistics for Windows Version 20.0.

## Results

### Main reasons for students’ medical school choice

#### Year-1

Of the Year-1 students, 95.2% (n=300) indicated one of the proposed reasons as the main reason (see Table 2). Fifteen Year-1 students (4.8%) provided ‘other’ reasons, of which two could be classified as ‘city’ and two could be classified as ‘curriculum’. Two reported more than one reason and were unclear as to what the main reason was. Examples of the other reasons were ‘having been treated in the university hospital’, ‘friends and family studying at the same university’ and ‘only option due to a late application’. Most students in Year-1 (56.9%, n=173) indicated the selection procedure as the main reason for their medical school choice, followed by city (24.7%, n=75), curriculum (11.2%, n=34) and university culture (7.0%, n=22).

#### Year-4

Of the Year-4 students, 86.5% (n = 141) indicated one of the proposed reasons as the main reason (see Table 2). Twenty-two Year-4 (13.5%) students indicated ‘other’ reasons, of which three could be classified as ‘city’ and one could be classified as ‘curriculum’. Four reported more than one reason and were unclear as to what the main reason was. Examples of other reasons were that students were already studying at that university or had the opportunity to skip a year at that specific medical school. Among Year-4 students, the results revealed a similar pattern. Almost half of the students 46.9% (n=68) based their medical school choice on the selection procedure, 36.0% (n=52) on the city, 12.4% (n=18) on the curriculum and 4.8% (n=7) on the university culture. Analyses were conducted using only the main categories ‘city’, ‘curriculum’, ‘university culture’ and ‘selection procedure’. Correlations between the reasons for students’ medical school choice and motivation variables are provided in the Appendix.

### Association between the reasons for students’ medical school choice and characteristics

#### Year-1

Binary regression analyses showed that city, curriculum, university culture and selection procedure as the main reasons for students’ medical school choice were associated with students’ characteristics (see Table 3). Females were more likely to have chosen based on the city than males (OR = 2.20, 95% CI 1.16–4.15, 0.02). Students at VUmc (OR = 2.43, 95% CI 1.23–4.79, p = 0.01) and AMC (OR = 3.43, 95% IC 1.66–7.06, p = 0.00) were more likely to have chosen based on the city than students at UMCG. First-generation university students were less likely to have chosen based on the curriculum than students whose parent(s) attended higher education (OR = 0.28, 95% CI 0.11–0.07, p = 0.01). Students at VUmc (OR = 3.11, 95% CI 1.19–8.16, p = 0.02) and AMC (OR = 3.46, 95% CI 1.19–10.03, p = 0.02) were more likely to have chosen based on the curriculum. Students at UMCG were more likely to have indicated the university culture as the main reason than AMC students (OR = 0.24, 95% CI 0.07-0.87, p = 0.03). Females (OR = 0.56, 95% CI 0.31–1.00, p = 0.05) and older students (OR = 1.69, 95% CI 1.00–2.84, p = 0.05) were more likely to have chosen based on the selection procedure.

#### Year-4

Binary regression analyses showed that the city, curriculum and selection procedure as the main reasons for medical school choice were associated with students’ characteristics. Students at VUmc (OR=4.72, 95% CI 1.58–14.15, p = 0.01) and AMC (OR=5.15, 95% CI 1.72–15.46, p = 0.00) were more likely to have indicated the city as the main reason for their medical school choice. Minority students, both Western (OR=0.12, 95% CI 0.02–0.60, p= 0.01) and non-Western (OR=0.07, 95% CI 0.01–0.74, p=0.03) were less likely to indicate the curriculum as the main reason for medical school choice than Dutch students. Students at VUmc (OR = 0.17, 95% CI 0.06–0.48, p = 0.00) and AMC (OR = 0.39, 95% CI 0.16–0.95, p = 0.04) were less likely to have indicated the selection procedure as the main reason than students at UMCG.

### Association between the reasons for students’ medical school choice and student motivation

#### Year-1

We report the overall scores for the Strength of motivation (M=55.5, SD=6.9), as well as the subscale scores, namely Willingness to sacrifice (M=17.5, SD=2.8), Readiness to start (M=18.7, SD=3.1) and Persistence (M=19.3, SD= 2.6), and the overall scores for Autonomous motivation (M = 4.3, SD=0.4) and Controlled motivation (M=2.0, SD=0.7). ANCOVAs yielded no associations between the main reason for students’ medical school choice and the Strength of motivation (F_(3,291)_=3.58, p=0.68), Willingness to sacrifice (F_(3,295)_=5.03, p=0.09), Readiness to start (F_(3,298) _=1.09, p= 0.09), Persistence (F_(3,294)_=2.66, p=0.05), Autonomous motivation (F_(3,292)_=1.58, p=0.20), and Controlled motivation (F_(3,293) _=1.63, p=0.18) see Table 4.

#### Year-4

We report the overall scores for the Strength of motivation (M=52.4, SD=6.2), Willingness to sacrifice (M=16.5, SD = 2.7), Readiness to start (M=17.4, SD=3.2), Persistence (M= 18.4, SD=2.5), Autonomous motivation (M=4.2, SD=0.4) and Controlled motivation (M=1.9, SD=0.7). ANCOVAs yielded no associations between the main reason for students’ medical school choice and the Strength of motivation (F_(3,135) _= 0.50, p=0.68), Willingness to sacrifice (F_(3,139) _= 2.36, p=0.07), Readiness to start (F_(3,137) _=0.44, p=0.73), Persistence (F_(3,137)_=0.54, p=0.66), Autonomous motivation (F_(3,137) _= 1.07, p=0.37), and Controlled motivation (F_(3,137) _=1.98, p =0.12).

**Table 4:** ANCOVA analyses of mean differences in motivation outcomes in the groups of students categorised by main reason for medical school choice

Statistics/Variable	N	Autonomous motivation^†^	Controlled motivation^†^	Strength of motivation^‡^	Willingness to sacrifice^¶^	Readiness to start^¶^	Persistence^¶^
Year-1		Mean (SE)^*^	Mean (SE)	Mean (SE)	Mean (SE)	Mean (SE)	Mean (SE)
City	75	4.22 (0.05)	1.94 (0.08)	53.97 (0.80)	16.64 (0.33)	18.32 (0.36)	18.96 (0.30)
Curriculum	34	4.38 (0.08)	1.88 (0.12)	58.54 (1.21)	18.79 (0.48)	19.46 (0.53)	20.43 (0.45)
University culture	22	4.18 (0.09)	2.22 (0.14)	54.69 (1.48)	17.45 (0.61)	18.65 (0.66)	18.96 (0.55)
Selection procedure	173	4.30 (0.03)	2.05 (0.05)	55.82 (0.52)	17.72 (0.22)	18.79 (0.24)	19.32 (0.20)
F-value by group		F_3, 292 _= 1.58	F_3, 293_ = 1.63	F_3, 291_ = 3.58	F_3, 295_ = 5.03	F_3, 298_ = 1.09	F_3, 294_ = 2.66
p-value		0.20	0.18	0.68	0.09	0.35	0.05
Year-4							
City	52	4.15 (0.06)	1.78 (0.09)	52.69 (0.87)	16.75 (0.38)	17.16 (0.44)	18.69 (0.34)
Curriculum	18	4.32 (0.10)	1.70 (0.16)	52.62 (1.46)	16.72 (0.64)	17.53 (0.75)	18.38 (0.58)
University culture	7	4.28 (0.15)	1.49 (0.25)	55.46 (2.53)	18.92 (1.02)	17.96 (1.30)	18.75 (0.92)
Selection procedure	68	4.15 (0.05)	1.97 (0.08)	52.24 (0.77)	16.17 (0.33)	17.81 (0.39)	18.14 (0.30)
F-value by group		F_3, 137_ = 0.07	F_3, 137_ = 1.98	F_3, 135_ = 0.50	F_3, 139_ = 2.36	F_3, 137_ = 0.44	F_3, 137_ = 0.54
p-value		0.37	0.12	0.68	0.07	0.73	0.66

*Corrected mean; SE = standard error; † Possible score range is 1 – 5; ‡ Possible score range is 15 – 75; ¶ Possible score range is 5 – 25.

## Discussion

In this study, we explored students’ main reasons for their medical school choice and whether these main reasons are associated with students’ characteristics and motivation for their medical study during the medical programme. Our study adds to the existing literature addressing medical school choice, as it appears to be the first study investigating medical school choice in relation to motivation outcomes during the medical study and in a unique setting in which students can only apply to one medical school.

### Main reason for students’ medical school choice

We found that the selection procedure and city were most often indicated as the main reason students chose to apply to a particular medical school.Our study suggests that applicants mainly choose which medical school to apply to based on the selection procedure and the city. The curriculum had only been a critical factor in medical school choice for approximately 10% of students. Year-1 and Year-4 students showed similar patterns, although the importance of the selection procedure seems to have increased over the years (the main reason for 46.9% of Year-4 students and 56.9% of Year-1 students). We found selection to be an important factor in medical school choice in a setting in which applicants can participate in selection at only one medical school per year. A medical school choice based on the selection procedure can be considered a strategic choice. Earlier research from the UK, where applicants have to limit their choice to four medical schools, also showed how applicants are focused on selection.^29^ Starting in 2017, medical school admissions in the Netherlands will be selection-based only, which raises doubts about achieving the desired student-curriculum fit. Besides providing proper information about the medical programme during recruitment activities, applying a selection procedure that reflects the curriculum may become even more important for both matching and selection purposes. Applicants can judge whether important curricular aspects appeal to them, while medical schools can assess which applicants have the most potential to perform well in their curriculum.

### Reasons for students’ medical school choice and students’ characteristics

Different types of students reported the city, curriculum, university culture or selection procedure as the main reason to varying extents. Medical school choice approaches varied across students with different background characteristics.  Among Year-1 students, female students in our study were more likely to have based their choice on the city than male students, while male students’ choices were more often based on the selection procedure. In other research, gender differences were found for curriculum aspects as a reason for students’ medical school choice.^6^ We did not find differences in this respect, which may be explained by the different study designs. In our study, students had to indicate one main reason, while in the UK study students were asked to indicate three reasons. Curriculum aspects may have been important to the males and females in our sample to varying extents as well, but clearly not as the main reason. Among Year-4 students, Dutch students were more likely to have taken the curriculum into account than both Western and non-Western minority students.

Students at the three medical schools differed in their approaches as well. Two medical schools in our study are situated in the same city, Amsterdam. Students from these two schools showed similar approaches. These approaches differed from those of students from the smaller city, Groningen. For the students from Amsterdam, the city and curriculum were more often important factors than for the students from Groningen. This raises the question of what students valued the most in the school of their choice after they decided to apply to a medical school in Amsterdam. The selection procedure was more often important to the Year-4 students from Groningen than to the students from Amsterdam. The selection ratio was equal to that of the other medical schools at the time these students had applied (i.e. 50%). The preference for the selection procedure used at UMCG among the students from Groningen was likely related to the content of the procedure. In that period, multiple mini interviews (MMIs), which were used at UMCG, were not commonly used in Dutch medical school selection. Other medical schools used predominantly academic tools to select their students. It is possible that this distinctive part of the UMCG procedure appealed to applicants.

This also raises the question of whether different types of selection procedures attract different types of students. Medical schools should realise that the approaches of different types of applicants can vary. This highlights the importance of recruitment strategies that focus on the types of students they seek.

### Reasons for students’ medical school choice and motivation during the medical study

The main reasons for students’ medical school choice were not associated with their motivation for their medical study during the medical programme. Although most students seem to enrol in medical study with a strategic approach, their motivation during their medical study is not inferior to the motivation of those who base their choice on more autonomous motivations, such as the curriculum. This suggests that the medical schools in the current study may have been successful in attracting students who are a good fit for their curriculum using their selection procedure. The strength of motivation among medical students reported in this study is comparable to findings from other studies^25^^,^^30^^,^^31^ and can be considered good. In addition, students reported more autonomous motivation and less controlled motivation than students from teacher training institutions in another study.^12^ Overall, the motivation of Year-4 students appears to be lower than the motivation of Year-1 students, which is in line with a previously reported reduction in students’ motivation throughout their medical study.^32^

### Limitations

Several limitations must be considered when interpreting the findings of this study. First, we are unsure whether our study sample is representative of the population with regards to the main reasons and motivation. The most motivated students may be more likely to participate in research, although the data did include reports of low strength and autonomous motivation and high controlled motivation. Second, the Year-4 students had to recall what their main reason for their medical school choice had been several years before. The reasons they have reported may not be accurate. Moreover, the educational programme may have influenced their motivation.^15^ To examine whether the motivation of students who enter their medical study with varying approaches develops differently throughout the study, a longitudinal design would be more appropriate. Lastly, while providing four specific reasons for students’ medical school choice in the questionnaire allowed a better assessment of associations with the other variables, this method clearly did not cover the full range of possible reasons and the nuances in the proposed reasons. However, because we based the provided reasons on previous findings from the literature^3^^-^^8^ and considering that the participants only provided several other reasons, we believe these reasons to be a fair representation of those of most applicants. In addition, we have not gathered information about *why* students chose a certain selection procedure. Therefore, we do not know whether their choice was mainly based on the criteria assessed or the tools used in the selection procedure or on the acceptance ratio of the medical school. The results of the current study led us to conduct a qualitative study to gain a better understanding of applicants’ pre-admissions behaviour.

## Conclusions

Most applicants strategically choose a specific medical school. Considering the importance of the selection procedure for applicants, our findings stress the importance of aligning of the selection procedure with the medical schools’ curriculum characteristics and values. This can involve including a lecture in which a specific theme, such as an area of the human body, is the basis for organizing the course material (when education is more theme-based) or a lecture in which a specific problem drives the acquisition of knowledge (when education is more problem-based), as well as using tests that are representative of assessment during students’ medical study.Different approaches are not associated with differences in students’ motivation during their medical study. Medical schools should, however, take into account the different approaches towards the medical school choice among applicants with various background characteristics if they desire to attract a student population that reflects the diversity of the patient population.

### Acknowledgements

We thank Francisca Galindo-Garre, PhD, and Marianne Jonker, PhD, from the Department of Epidemiology and Biostatistics, VU University Medical Center, for their help with the statistical analyses. 

### Conflict of Interest

The authors declare that they have no conflict of interest.

## Supplementary material

Supplementary file 1 Appendix. Pearson correlations between reasons for medical school choice and motivation outcomes
