# Supplementary material for: Students’ approaches to medical school choice: relationship with students’ characteristics and motivation
Source: Int J Med Educ. 2017 Jun 12;8:217–26. doi: 10.5116/ijme.5921.5090 (PMC5511747; doi:10.5116/ijme.5921.5090)
Supplement: Supplementary file 1 — Appendix. Pearson correlations between reasons for medical school choice and motivation outcomes [file ijme-8-217-S1.pdf]

Appendix

Pearson correlations between reasons for medical school choice and motivation outcomes

Year-1 / Year-4	1 Reason City	2 Reason Curriculum	3 Reason University culture	4 Reason Selection procedure	5 Autonomous motivation	6 Controlled motivation	7 Strength of motivation	8 Willingness to sacrifice	9 Readiness to start	10 Persistence
1 Reason City	-	n/a	n/a	n/a	-0.085	-0.058	**-0.140***	**-0.185****	-0.092	-0.078
2 Reason Curriculum	n/a	-	n/a	n/a	0.067	-0.091	**0.132***	**0.141***	0.067	**0.137***
3 Reason University culture	n/a	n/a	-	n/a	-0.071	0.078	-0.046	-0.017	-0.022	-0.045
4 Reason Selection procedure	n/a	n/a	n/a	-	0.069	0.067	0.063	0.079	0.049	0.006
5 Autonomous motivation	-0.048	0.123	0.052	-0.058	-	-0.017	**0.572****	**0.459****	**0.524****	**0.395****
6 Controlled motivation	-0.075	-0.086	-0.125	**0.184***	-0.042	-	-0.055	-0.076	-0.007	-0.047
7 Strength of motivation	0.000	-0.022	0.081	-0.018	**0.407****	-0.100	-	**0.846****	**0.820****	**0.746****
8 Willingness to sacrifice	0.036	-0.003	**0.185***	-0.112	**0.247****	-0.067	**0.745****	-	**0.547****	**0.497****
9 Readiness to start	-0.094	-0.012	0.019	0.091	**0.386****	-0.072	**0.752****	**0.281****	-	**0.374****
10 Persistence	0.069	-0.027	0.029	-0.061	**0.222****	-0.108	**0.700****	**0.390****	**0.254****	-

**Correlation is significant at the 0.01 level (2-tailed)
*Correlation is significant at the 0.05 level (2-tailed)
Blue cells represent Year-1 correlations and white cells represent Year-4 correlations
n/a = not available
